# Accreditation in a Sub Saharan Medical School: a case study at Makerere University

**DOI:** 10.1186/1472-6920-13-73

**Published:** 2013-05-24

**Authors:** Moses Galukande, Kenneth Opio, Noeline Nakasujja, William Buwembo, Stephen C Kijjambu, Shafik Dharamsi, Sam Luboga, Nelson K Sewankambo, Robert Woollard

**Affiliations:** 1 Department of Surgery, College of Health Sciences, Makerere University, Kampala, Uganda; 2 Department of Medicine, College of Health Sciences, Makerere University, Kampala, Uganda; 3 Department of Psychiatry, College of Health Sciences, Makerere University, Kampala, Uganda; 4 Department of Anatomy, College of Health Sciences, Makerere University, Kampala, Uganda; 5 Department of Family Practice, Faculty of Medicine, University of British Columbia, Vancouver, BC, Canada

**Keywords:** Accreditation, Standards, Medical education

## Abstract

**Introduction:**

Of more than the 2,323 recognized and operating medical schools in 177 countries (world wide) not all are subjected to external evaluation and accreditation procedures. Quality Assurance in medical education is part of a medical school’s ethical responsibility and social accountability. Pushing this agenda in the midst of resource limitation, numerous competing interests and an already overwhelmed workforce were some of the challenges faced but it is a critical element of our medical profession’s social contract. This analysis paper highlights the process of standard defining for Medical Education in a typically low resourced sub Saharan medial school environment.

**Methods:**

The World Federation for Medical Education template was used as an operating point to define standards. A wide range of stakeholders participated and meaningfully contributed in several consensus meetings. Effective participatory techniques were used for the information gathering process and analysis.

**Results:**

Standards with a clear intent to enhance education were set through consensus. A cyclic process of continually measuring, judging and improving all standards was agreed and defined. Examples of the domains tackled are stated.

**Conclusion:**

Our efforts are good for our patients, our communities and for the future of health care in Uganda and the East African region.

## Introduction

Not all of the 2,323 recognised and operating medical schools worldwide are subject to external evaluation and accreditation procedures [1]. Quality assurance in medical education is part of every medical school’s ethical responsibility and social accountability [2-4]. Simply put, physicians graduating from any particular medical school must be trained to provide high quality medical care within clearly defined criteria of minimally accepted standards [5]. Makerere University College of Health Sciences, a 90 year old institution, until recently had not explicitly defined minimum standards as an approach to training health care providers.

The World Health Organization (WHO) and the World Federation for Medical Education (WFME) Strategic Partnership to improve medical education was formed in 2004 to support the development of quality assurance mechanisms and related standards for improving medical education worldwide [6-8]. In response, the College of Health Sciences at Makerere University em barked on a major curriculum renewal process. Teaching and learning methods moved from traditional, teacher-centred education to one that is fundamentally student-centred. The use of problem based learning (PBL), small group and collaborative learning models as well as community-based education and service have formed the key pedagogical elements of medical education at Makerere [9-11]. A Quality Assurance (QA) Task Force was established to embark on the process of developing a mechanism for total quality management, including defining standards for medical education.

The purpose of this paper is not only to describe the process and rationale of standards definition but demonstrate a translation of WFME standards into practice in a resource-limited context. Examples rather than the entire list of standards defined is cited in here.

## Methods and materials

In keeping with Makerere’s commitment to social accountability [12] as defined by TUFH a WHO associated organisation [2], the development of quality improvement standards involved a process of wide consultation with various stakeholders from within and outside the College of Health Sciences, including the Ministry of Health, the various councils: Medical and Dental, Nursing, Pharmacy and Allied professions. The others were student representations, administrators, policy makers, representatives from the Makerere University Quality Assurance body, lecturers and the National Council for Higher Education. The Task Force consulted also with various individuals from outside the country such as medical faculty from The University of British Columbia, Canada, Nuffic project consultants from the Netherlands and Kenya. The Makerere QA unit is charged with over seeing the definition of standards, establishment of a frame work to support execution and monitor compliance. All in the quest of providing the highest possible quality of education at Makerere.

The process costs were kept low and the attendance high by way of running several half- day non-residential, a strategy preferred to full days by stakeholders, so that they could utilize the remaining half of the day to attend to other often pressing matters. A non residential workshop saves on otherwise high hotel bills. Participatory Question Based Facilitation (PQBF) and Visualization in Participatory Planning (VIPP) approaches were used to generate and sustain individual interest [13-16]. Relevant materials were sent to the participants prior to the meetings.

The participatory Question Based Facilitation grows directly out of the need to improve planning process in situations, which are entrenched or stagnated, highly competitive or conflictual. It is based on participatory techniques designed to diffuse tensions, tackle core problems, generate relevant solutions, enhance commitment and create a culture effective teamwork. This approach refers to a creative combination of different approaches to planning, centred around professional facilitation based on questions [14,17].

The Task Force participated in four workshops, weekly quality assurance meetings, and an international visit to study an accreditation process in Canada. Collaborators experienced in quality assurance processes from The University of British Columbia, Vancouver, Canada, Groningen University, and a Nuffic/Netherlands Quality Assurance consultant from Kenya facilitated two of the workshops; and senior academic staff from Makerere facilitated two workshops.

The WFME template [18] was used as a starting point to define standards for Makerere. The Task Force examined the Template in significant detail and reached a consensus on what ought to be adopted. Three working groups were formed with an external expert facilitator for each group. A SWOT analysis of the medical school resulted in the development of the said standards using the participatory Approaches including PQBF and VIPP [13,14]. A detailed report was presented to the Makerere Faculty Educational Committee for review, following which it was submitted to the then Faculty Board for approval. Consensus is deemed reached after an overwhelming vote on an issue at hand and or hearing out and convincing the last doubtful voice. Subjecting the process to debates is presumed to generate ownership of the standards among stakeholders.

## Results

During the initial workshops, the Faculty and its partners formed consensus on two important aspects of the WFME approach to the development of standards and systems [19]; First, the idea of distinguishing between “Basic” and “Quality Improvement” levels of compliance made intuitive sense as we struggled with aspiring to excellence in a very low resource situation. By reaching consensus on what must be we could then hatch a common dream on what should be.

The second valuable insight gained from WFME standards was the achievement of consensus on the nine “domains” in which our standards should nest (see Table 1). The four-day exercise whereby we explored, refined, and reached a general consensus on their meaning in the context of Uganda provided an invaluable foundation and helped cement the relationships within the primary stakeholders required for our ultimate success. The context of Ugandan here refers to what the needs of the country are, the mission of the college and the unique challenges of constrained human resource educational materials and finances.

**Table 1:** Showing the WFME standards domains

	**Domains**
1	Mission and Objectives of Medical School
2	Educational Programme
3	Assessment of Students
4	Students
5	Academic Staff/Faculty
6	Educational Resources
7	Programme Evaluation
8	Governance and Administration
9	Continuous Renewal

## Engagement of partners

**Figure 1:**
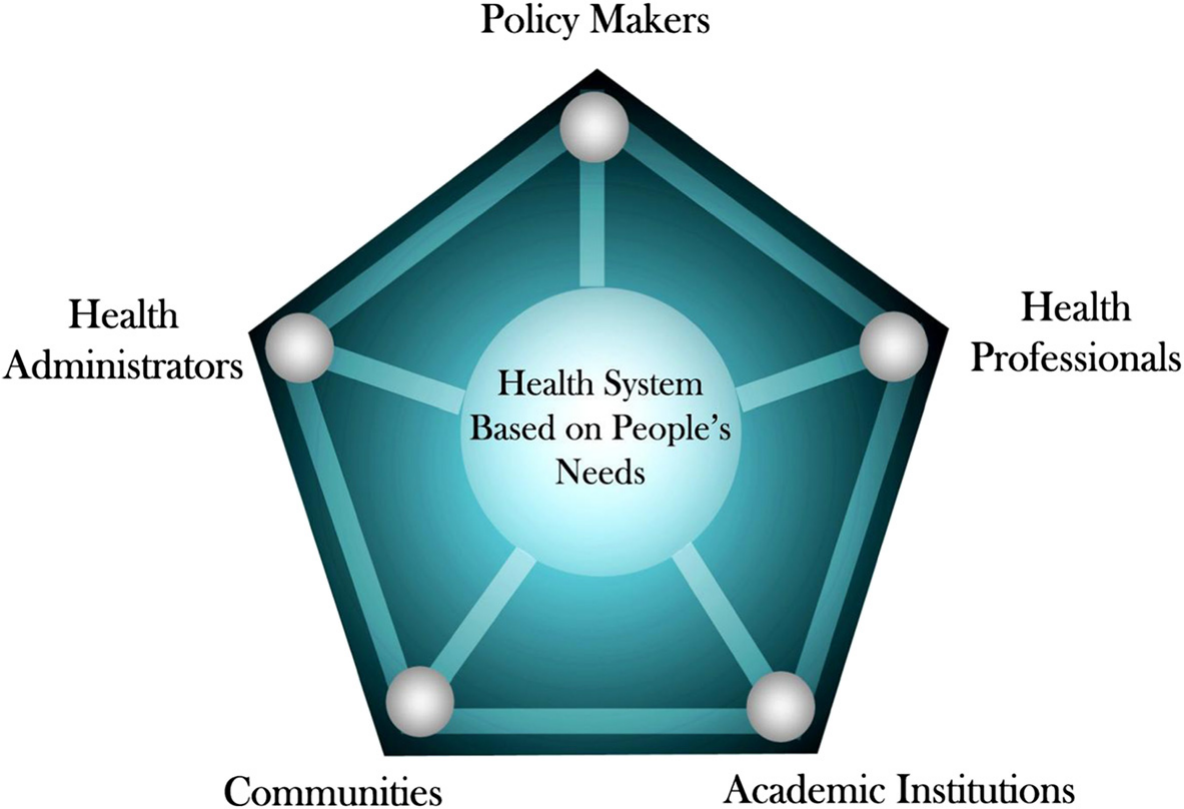
Partnership pentagram.

The “partnership pentagram” outlined by the World Health Organization in their ground breaking initiative “Towards Unity for Health for All (TUFH) [2], and illustrated in Figure 1, was used as the working model to inform the development of partnerships required for an effective accreditation system in Uganda that would focus on the needs of the citizens of Uganda and East Africa. This perspective has been used to good effect elsewhere [3,6,7,20,21] and was instrumental in our engagement of the other sectors and professions in Uganda. This was instrumental in the sense that the choice of stakeholders who engaged this process was informed by the knowledge of the partnership pentagram.

Through the use of an appreciative approach [22] we were able to engage a broad range of people and institutions in the refinement of standards in each of the “domains” and to establish mutual trust in our common future – this list represents a robust consensus and a commitment on the part of Makerere to hold itself to these standards (examples of which are shown in Table 2) and to help in the development of an inter-institutional system of quality assurance/improvement in Uganda and East Africa. This process of engagement of stakeholders was an essential part of success to this point. While they served as an invaluable template, several of the WFME standards benefited from adjustments to standards by those participating in the workshops. At the same time, the process of achieving consensus on these modifications helped to develop the partnerships in the ‘pentagram” to a level that bodes well for future joint action to achieve a SYSTEM of accountable accreditation devoted to quality improvement.

**Table 2:** Showing specific examples of standards and the domains they belong to

**Domain 1: Participation in formulation of mission and objectives**	
Basic standard (Level I)	1. The mission statement and objectives of a medical school must be defined by its principal stakeholders
Quality development (Level II)	2. Formulation of mission statements and objectives should be based on input from a wider range of stakeholders.
**Academic autonomy**	
Basic standard (Level I)	3. There must be a policy for which the administration and faculty/academic staff of the medical school are responsible, within which they have freedom to design the curriculum and allocate the resources necessary for its implementation.
Quality development (Level II)	4. The contributions of all academic staff should address the actual curriculum and the educational resources should be distributed in relation to the educational needs.
**Domain 2: Educational outcome**	
Basic standard (Level I)	5. The medical school shall define competencies that students should exhibit on graduation in relation to their subsequent training and future roles in the health system.
Quality development (Level II)	6. The linkage of competencies to be acquired by graduation with that to be acquired in postgraduate training should be specified.
	7. Measures of, and information about, performances of the graduates should be used as feedback to programme development.
**Linkage with Medical practice and the Health Care system**	
Basic standard (Level I)	8. Operational linkage must be assured between the educational programme and the subsequent stage of training or practice that the student will enter after graduation.
Quality development (Level II)	9. The curriculum committee should seek input from the environment in which graduates will be expected to work and should undertake programme modification in response to feedback from the community and society regularly.
**Domain 4: Students Admission policy and selection**	
Basic standard (Level I)	10. The medical school must have an admission policy including a clear statement on the process of selection of students.
Quality development (Level II)	11. The admission policy should be reviewed periodically, based on relevant societal and professional data, to comply with the social responsibilities of the institution and the health needs of community and society.
	12. The relationship between selections, the educational programme and desired qualities of graduates should be stated.
**Student intake**	
Basic standard (Level I)	13. The size of student intake must be defined and related to the capacity of the medical school at all stages of education and training.
Quality development (Level II)	14. The size and nature of student intake should be reviewed in consultation with relevant stakeholders and regulated periodically to meet the needs of community and society.
**Domain 5: Academic Staff/Faculty Recruitment policy**	
Basic standard (Level I)	15. There must be a staff recruitment policy, which outlines the type, responsibilities and balance of academic staff required to deliver the curriculum adequately, including the balance between medical and non-medical academic staff, support staff, Technical staff and between full-time and part-time staff.
	16. The policy shall address issues of gender balance.
	17. The responsibilities of the staff shall be explicitly specified job description and monitored.
	18. Efforts shall be made for the policy to be well understood by the faculty.
	19. Clarity of duties as regards Ministry of Health and Ministry of Education shall be explicitly documented.
Quality development (Level II)	20. A policy should be developed for staff selection criteria, including scientific, educational and clinical merit, relationship to the mission of the institution, economic considerations and issues of local significance.
	21. There should be a staff retention policy, which outlines strategies to prevent and/or minimize staff brain drain.
**Domain 6: Program Evaluation Involvement of stakeholders**	
Basic standard (Level I)	22. Programme evaluation must involve the governance and administration of the medical school, the academic staff, the students and the public.
Quality development (Level II)	23. A wider range of stakeholders should have access to results of course and programme evaluation, and their views on the relevance and development of the curriculum should be considered.
	24. There should be a Faculty level tracer study of graduates.
	25. There should be community representation at faculty level to meet social responsibility obligations.
**Domain 8: Government and Administration Interaction with health sector**	
Basic standard (Level I)	26. The medical school must have a constructive interaction with the health and health-related sectors of society and government.
Quality development (Level II)	27. The collaboration with partners of the health sector should be formalised.
	28. There should be a resource mobilization office, as a major faculty activity.
	29. The Medical School should develop an effective system of communication for students, staff and the general public.
**Communication**	
	30. A Public Relations role should be defined and a host office named.
**9. Continuous Renewal**	
Basic standard (Level I)	31. The medical school must, as a dynamic institution, initiate procedures for regular reviewing and updating of its structure and functions and must rectify documented deficiencies.
	32. There must be a major review of Curriculae every 10 years
Quality development (Level II)	33. The process of renewal should be based on prospective studies and analyses and should lead to the revisions of the policies and practices of the medical school in accordance with past experience, present activities and future perspectives.

## Discussion

These standards are concerned with categories of the content, process, educational environment and outcome of medical education. They are to function as a lever for change and reform. Compliance with these standards is a concern of all stakeholders. Compliance is a pre requisite to realizing the intended change and reform.

These standards are intended not only to set minimum requirements but also to encourage quality improvement beyond the minimum. Levels I and II in the standards document offer a description of what must be followed by a statement of what should be, resources permitting.

Assuring and enhancing the quality of teaching and learning in Universities is currently of major concern. In this era of audit and accountability, there is an imperative to demonstrate and document that appropriate standards have been set and maintained in professional education [23].

### Context

Whereas many countries have established national systems for the assessment of quality in higher education [24] some under-resourced countries, such as Uganda, have not fully developed such systems. For the College of Health Sciences at Makerere to undertake the process of defining its own standards is in one way filling the void that is yet to be filled by the national system in another way it is stimulating and contributing to the full development of such a system. The National Councils will perhaps adopt and work with what is already in place. Whereas for more than seven decades the College of Health Sciences at Makerere was the sole trainer for degree health workers in Uganda, the last decade has seen the addition of five institutions. This state of affairs begs for the definition of uniform standards upon which all should work and be judged.

The WFME template was found to be comprehensive in content, however, there were challenges getting consensus for every aspect. This was overcome by using participatory planning techniques [15]. Where everyone’s idea, voice was heard and considered. Identifiable streams of the processes contribute to the participatory approaches employed and are briefly outlined here: Paulo Freire’s concretisation movement, which emphasizes awareness raising and empowerment [12]. Experiential learning associated with Orlando Fals Borda of Colombia which emphasizes multi dimensional thinking (cognitive), feeling (affective) and acting (psychomotor) [1].

Visualization techniques originating from the Quick born Team of Germany associated with Eberhard Schrelle and his colleagues who designed training in which decision makers and those solutions together, resulting in common action [17].

Visualization in Participatory programs which was developed in the early 1990s by a team led by Neill Mckee. Mckee had learned a variety of participatory techniques from Hermann Tillmann and Maruja Salas, which he introduced into the planning processes for social mobilization and communication in CMCEF programmes in Bangladesh [16].

### Stakeholders’ representation & engagement

The challenge of ownership and acceptance was tackled by ensuring wide representation from stakeholders. Adequate notice was given, easily accessible and convenient venue chosen.

The challenge of generating interest and allocating time for this activity in light of other activities competing for time and resources was tackled by, a sustained campaign of dissemination of information about a forth coming meeting and its rationale. A ‘pilot’ or test run meet was called to gauge participation and interest before the ‘big’ one came. The issues of a champion who would consistently run with this project was raised, debated and agreed.

### Implementation

The perception of defining standards would generate extra work for faculty became apparent. The concerns being more work with no matching compensation for time and effort. The response to this challenge was in the promise of making standards, relate to work routine and embedded in the institutional culture. Routinizing this work would spread it out institution wide, lessening, improving individual load, communication and hopefully job satisfaction.

## Conclusions

Defining standards is not an end in itself. They must be used in a SYSTEM of accreditation that enables institutional self-reflection and institutional peer review to hold the medical schools mutually responsible for the continuous improvement of medical education in Uganda-and perhaps East Africa. Acceptance and compliance by all stakeholders will support this effort. When the system is built and the standards used in action, there is a clear intent to enhance education and to link that in turn to enhanced practice and health outcomes. The cyclic process of continually measuring, judging and improving all stated aspects of the system has begun with the process of establishing the standards in the way we have described above.

## Abbreviations

WHO: World Health Organization; WFME: World Federation for Medical Education; PBL: Problem based learning; PQBF: Participatory question based facilitation; VIPP: Visualization in participatory planning; TUFH: Towards unity for health for all; QA: Quality assurance.

## Competing interests

The authors declare no competing interests.

## Author contributions

MS and KO originated the concept. MG, KO, NN, WB, SCK, SD, SL, RW collected data MG wrote first draft. NKS gave intellectual input. All authors reviewed drafts and approved final manuscript.

## Pre-publication history

The pre-publication history for this paper can be accessed here:

http://www.biomedcentral.com/1472-6920/13/73/prepub
